# Fasting time and lipid parameters: association with hepatic steatosis — data from a random population sample

**DOI:** 10.1186/1476-511X-13-18

**Published:** 2014-01-22

**Authors:** Martin Gruchot, Tilmann Graeter, Suemeyra Oeztuerk, Mark Martin Haenle, Wolfgang Koenig, Armin Imhof, Bernhard Otto Boehm, Richard Andrew Mason, Wolfgang Kratzer, Atilla Serif Akinli

**Affiliations:** 1 Department of Internal Medicine I, University Hospital Ulm, Albert-Einstein-Allee 23, 89081 Ulm, Germany; 2 Department of Diagnostic and Interventional Radiology, University Hospital Ulm, Albert-Einstein-Allee 23, 89081 Ulm, Germany; 3 Department of Internal Medicine II - Cardiology, University Hospital Ulm, Albert-Einstein-Allee 23, 89081 Ulm, Germany; 4 Louis Stokes Cleveland Department of Veterans Affairs Medical Center, 10700 East Boulevard, Cleveland, OH 44106 USA

**Keywords:** Lipids, Total cholesterol, LDL cholesterol, HDL cholesterol, Triglycerides, Fasting time, Population-based, Cross-sectional, Non-alcoholic fatty liver disease (NAFLD)

## Abstract

**Background:**

Current guidelines recommend measuring plasma lipids in *fasting* patients. Recent studies, however, suggest that variation in plasma lipid concentrations secondary to fasting time may be minimal. Objective of the present study was to investigate the impact of fasting time on plasma lipid concentrations (total cholesterol, HDL and LDL cholesterol, triglycerides). A second objective was to determine the effect of non-alcoholic fatty liver disease exerted on the above-mentioned lipid levels.

**Method:**

Subjects participating in a population-based cross-sectional study (2,445 subjects; 51.7% females) were questioned at time of phlebotomy regarding duration of pre-phlebotomy fasting. Total cholesterol, LDL and HDL cholesterol, and triglycerides were determined and correlated with length of fasting. An upper abdominal ultrasonographic examination was performed and body-mass index (BMI) and waist-to-hip ratio (WHR) were calculated. Subjects were divided into three groups based on their reported fasting periods of 1–4 h, 4–8 h and > 8 h. After application of the exclusion criteria, a total of 1,195 subjects (52.4% females) were included in the study collective. The Kruskal-Wallis test was used for continuous variables and the chi-square test for categorical variables. The effects of age, BMI, WHR, alcohol consumption, fasting time and hepatic steatosis on the respective lipid variables were analyzed using multivariate logistic regression.

**Results:**

At multivariate analysis, fasting time was associated with elevated triglycerides (p = 0.0047 for 1–4 h and p = 0.0147 for 4–8 h among females; p < 0.0001 for 1–4 h and p = 0.0002 for 4–8 h among males) and reduced LDL cholesterol levels (p = 0.0003 for 1–4 h and p = 0.0327 for 4–8 h among males). Among males, hepatic steatosis represents an independent factor affecting elevated total cholesterol (p = 0.0278) and triglyceride concentrations (p = 0.0002).

**Conclusion:**

Total and HDL cholesterol concentrations are subject to slight variations in relation to the duration of the pre-phlebotomy fasting period. LDL cholesterol and triglycerides exhibit highly significant variability; the greatest impact is seen with the triglycerides. Fasting time represents an independent factor for reduced LDL cholesterol and elevated triglyceride concentrations. There is a close association between elevated lipids and hepatic steatosis.

## Background

The current guidelines (2001) of the National Cholesterol Education Program (NCEP) recommend that measurement of serum lipids, especially of LDL cholesterol and triglycerides, be performed in fasting patients [1]. The objective is to minimize individual variations in lipid levels, especially of LDL cholesterol and triglycerides, and to ensure that patients’ metabolic condition at the time of phlebotomy is comparable [2,3].

However, a few studies, some of them in large subject collectives, that investigated variations in lipid concentrations in relation to the duration of subjects’ fasting period, observed only slight variation and came to the conclusion that fasting is not an absolute requirement for measurement of HDL, LDL and total cholesterol [4-7]. Two current population-based studies have also found that measurement of serum lipids in non-fasting subjects differed less than had been previously expected from findings in those who were fasting at the time of phlebotomy [2,8]. Triglycerides show the greatest variability in relation to the fasting period [2,4,6,8]. Some studies, however, suggest that the predictive value of non-fasting triglyceride levels for cardiovascular disease is comparable to [8,9] or even superior to that of concentrations determined in fasting subjects [6,10,11]. Because humans normally are in a non-fasting condition during much of the day, it might be argued that lipid measurements obtained in a non-fasting subject are in fact more representative of the current metabolic status and may provide better evidence for potential disorders of postprandial lipid metabolism [1,2,9,12,13].

With these considerations in mind, we investigated the effects of the duration of pre-phlebotomy fasting time as well as other factors, such as age, body-mass index (BMI), waist-to-hip ratio (WHR) and alcohol consumption of the plasma concentrations of total cholesterol, HDL and LDL cholesterol, and triglycerides in a population-based subject collective. In addition, we assessed the association of lipid concentrations with the prevalence of hepatic steatosis.

## Results

A total of 1,195 subjects were included in the present study. Depending on the duration of their pre-phlebotomy fasting period, subjects were divided into three groups, with 151 subjects being included in the group reporting 1–4 hours’ fasting, 866 subjects in the group reporting 4–8 hours’ fasting and 178 subjects reporting having fasted for > 8 hours. An overview of anthropometric data for the three fasting groups is given in Table 1*.*

**Table 1:** Anthropometric data for the three fasting groups

	**Fasting groups**			**Total**
	**1-4 h**	**4-8 h**	**> 8 h**	
**Subjects (n, %)**	151 (12.6%)	866 (72.5%)	178 (14.9%)	1195
**Women (n, %)**	83 (55%)	472 (54.5%)	71 (39.9%)	626 (52.4%)
**Age (M ± SD) [years]**	38.2 ± 12.5	41.8 ± 12.4	38.2 ± 11.2	40.8 ± 12.3
**BMI (M ± SD) [kg/m**^**2**^**]**	24.9 ± 4.5	25.2 ± 4.5	25.6 ± 4.7	25.2 ± 4.6
**WHR (M ± SD)**	0.8 ± 0.1	0.8 ± 0.1	0.8 ± 0.1	0.8 ± 0.1
**NAFLD (n, %)**	33 (21.9%)	207 (23.9%)	39 (21.2%)	279 (23.4%)
**MetS (n, %)**	8 (5.3%)	30 (3.5%)	2 (1.1%)	40 (3.4%)
**Diabetes (n, %)**	3 (2%)	14 (1.6%)	3 (1.7%)	20 (1.7%)
**Alcohol consumption (n, %)**				
0 g/d	8 (5.3%)	23 (2.7%)	7 (3.9%)	38 (3.2%)
1-20 g/d	63 (41.7%)	292 (33.7%)	82 (46.1%)	437 (36.5%)
21-40 g/d	71 (47%)	465 (53.7%)	66 (37.1%)	602 (50.4%)
>40 g/d	9 (6%)	86 (9.9%)	23 (12.9%)	118 (9.9%)

BMI = body-mass index, d = day, g = gram, h = hour, kg = kilogram, m^2^ = square meter, MetS = metabolic syndrome, M = mean, n = number, NAFLD = non-alcoholic fatty liver disease, SD = standard deviation, WHR = waist-to-hip Ratio.

In males, there was significant variability between fasting groups in terms of the mean concentrations of *total cholesterol* (p = 0.0107), *LDL cholesterol* (p < 0.0001) and *triglycerides* (p < 0.0001). The respective means of the lipid fractions varied in relation to the means of the group reporting > 8 hours’ fasting by a maximum −5.6%, +7.7%, –17.1% and +46.2% with respect to *total cholesterol*, *HDL cholesterol*, *LDL cholesterol* and *triglycerides,* respectively (*see* Table 2). Multivariate analysis examined the effect on lipid values of age, BMI, WHR, alcohol consumption, hepatic steatosis and fasting time as independent factors. Here, a significant association was observed between *fasting time, LDL* c*holesterol* and *triglycerides* (*see* Table 3). The Odds Ratio (OR) for *elevated triglycerides* stood at 4.477 (*95% confidence interval [CI]*: 2.183 - 9.183, p < 0.0001) for the group reporting 1–4 hours’ fasting and 2.816 (*95% CI:* 1.634 - 4.854, p = 0.0002) for the group reporting 4–8 hours’ fasting. For *elevated LDL cholesterol,* the OR stood at 0.284 (*95% CI:* 0.143 - 0.565, p = 0.0003) and 0.588 (*95% CI:* 0.361 - 0.957, p = 0.0327) for the groups reporting 1–4 and 4–8 hours’ fasting, respectively.

**Table 2:** Mean concentrations of serum lipid fractions in the fasting groups and sexes

	**Women (n = 626)**		**Men (n = 569)**		**Total (n = 1195)**		**p**
	**n**	**M ± SD (%**^**§**^**)**	**n**	**M ± SD (%**^**§**^**)**	**n**	**M ± SD (%**^**§**^**)**	
**Total cholesterol**							
1-4 h	83	5.5 ± 1.0 (0%)	68	5.1 ± 0.9 (−5.6%)	151	5.3 ± 1.0 (−1.9%)	0.0179*
4-8 h	472	5.5 ± 1.0 (0%)	394	5.5 ± 1.1 (+1.9%)	866	5.5 ± 1.0 (+1.9%)	0.9079
> 8 h	71	5.5 ± 1.0 (Ref.)	107	5.4 ± 1.1 (Ref.)	178	5.4 ± 1.1 (Ref.)	0.6270
p		0.8198		0.0107*		0.0351*	
**LDL cholesterol**							
1-4 h	83	3.1 ± 0.8 (−6.1%)	68	2.9 ± 0.8 (−17.1%)	151	3.0 ± 0.8 (−11.8%)	0.0774
4-8 h	472	3.2 ± 0.9 (−3%)	394	3.3 ± 1.0 (−5.7%)	866	3.3 ± 0.9 (−2.9%)	0.0451*
> 8 h	71	3.3 ± 1.0 (Ref.)	107	3.5 ± 1.0 (Ref.)	178	3.4 ± 1.0 (Ref.)	0.3181
p		0.2460		<0.0001*		<0.0001*	
**HDL cholesterol**							
1-4 h	83	1.7 ± 0.4 (0%)	68	1.4 ± 0.3 (+7.7%)	151	1.6 ± 0.4 (+6.7%)	<.0001*
4-8 h	472	1.8 ± 0.4 (+5.9%)	394	1.4 ± 0.4 (+7.7%)	866	1.6 ± 0.4 (+6.7%)	<.0001*
> 8 h	71	1.7 ± 0.4 (Ref.)	107	1.3 ± 0.3 (Ref.)	178	1.5 ± 0.4 (Ref.)	<.0001*
p		0.1439		0.1818		0.0015*	
**Triglycerides**							
1-4 h	83	1.5 ± 1.0 (+66.7%)	68	1.9 ± 1.0 (+46.2%)	151	1.7 ± 1.0 (+54.5%)	0.0007*
4-8 h	472	1.2 ± 0.6 (+33.3%)	394	1.8 ± 0.9 (+38.5%)	866	1.5 ± 0.8 (+36.4%)	<.0001*
> 8 h	71	0.9 ± 0.4 (Ref.)	107	1.3 ± 0.7 (Ref.)	178	1.1 ± 0.6 (Ref.)	0.0009*
p		0.0002*		<0.0001*		<0.001*	

h = hour, HDL cholesterol = high-density lipoprotein cholesterol, LDL cholesterol = low-density lipoprotein cholesterol, M = mean, n = number, Ref. = reference range, SD = standard deviation.

*Statistical significance for p < 0.05.

^§^Percent variability in comparison with the > 8 hour fasting group.

**Table 3:** Association between fasting time and elevated lipid concentrations

	**Women**		**Men**	
	**OR (95%-CI)**	**p**	**OR (95%-CI)**	**p**
**Total cholesterol**				
1-4 h	1.180 (0.586 - 2.374)	0.6428	0.668 (0.342 - 1.306)	0.2382
4-8 h	0.941 (0.545 - 1.624)	0.8262	1.258 (0.786 - 2.014)	0.3383
> 8 h	Ref.	-	Ref.	-
**LDL cholesterol**				
1-4 h	0.948 (0.477 - 1.882)	0.8785	0.284 (0.143 - 0.565)	0.0003*
4-8 h	0.916 (0.533 - 1.574)	0.7499	0.588 (0.361 - 0.957)	0.0327*
> 8 h	Ref.	-	Ref.	-
**HDL cholesterol**				
1-4 h	1.453 (0.388 - 5.441)	0.1903	0.480 (0.199 - 1.161)	0.1036
4-8 h	1.235 (0.408 - 3.738)	0.3612	0,734 (0.414 - 1.301)	0.2900
> 8 h	Ref.	-	Ref.	-
**Triglycerides**				
1-4 h	9.190 (1.974 -42.776)	0.0047*	4.477 (2.183 - 9.183)	<0.0001*
4-8 h	6.114 (1.428 - 26.180)	0.0147*	2,816 (1.634 - 4.854)	0.0002*
> 8 h	Ref.	-	Ref.	-

95%-CI = 95% confidence interval, h = hour, HDL cholesterol = high-density lipoprotein cholesterol, LDL cholesterol = low-density lipoprotein cholesterol, OR = Odds-Ratio, Ref. = reference range.

*Significant association at p < 0.05.

Among women, significant variability related to the length of pre-phlebotomy fasting was observed only for *triglycerides* (*1*–*4 h:* 1.5 ± 1.0 mmol/l; *4*–*8 h:* 1.2 ± 0.6 mmol/l; *> 8 h:* 0.9 ± 0.4 mmol/l, p = 0.0002). The means of the lipid fractions in females varied in comparison with the average of the group reporting > 8 hours’ fasting by a maximum +5.9%, -6.1% and +66.7% for *HDL cholesterol*, *LDL cholesterol* and *triglycerides,* respectively. For total cholesterol, there was no variability between groups in relation to subjects’ pre-phlebotomy fasting period (Table 2)*.* Multivariate analysis demonstrated a significant association between *fasting time* as an independent and *triglycerides* as a dependent variable (Table 3)*.* The OR for *elevated triglycerides* stood at 9.190 (*95%-CI:* 1.974 - 42.776, p = 0.0047) for the group reporting 1–4 hours’ fasting and 6.114 (*95% CI:* 1.428 - 26.180, p = 0.0147) for the group reporting 4–8 hours’ fasting.

The prevalence of non-alcoholic fatty liver disease (NAFLD) in the total collective stood at 23.4% (n = 279), of whom 67% were men (p < 0.0001). A comparison of the anthropometric data for subjects with and without hepatic steatosis are given in Table 4*.* Subjects with fatty liver were, on average, older, had a higher average WHR and a higher mean BMI. Fully 74.6% of subjects with hepatic steatosis had an elevated WHR, while 47.7% had a BMI of 25–30 kg/m^2^ and 39.4% had a BMI of > 30 kg/m^2^ (p < 0.0001 for both groups).

**Table 4:** Anthropometric data in subjects with and without hepatic steatosis

	**Steatosis no**	**Steatose yes**	**Total**	**p**
	**(n = 916)**	**(n = 279)**	**(n = 1195)**	
***Anthropometric data***				
**Sex (n, %)**				
Female	534 (58.3%)	92 (33%)	626 (52.4%)	<0.0001*
Male	382 (41.7%)	187 (67%)	569 (47.6%)	
**Age (M ± SD) [years]**	38.8 ± 11.9	47.3 ± 11.7	40.8 ± 12.3	<0.0001*
**BMI (M ± SD) [kg/m**^**2**^**]**	24.0 ± 3.7	29.4 ± 4.6	25.2 ± 4.6	<0.0001*
**WHR (M ± SD)**	0.8 ± 0.1	0.9 ± 0.1	0.8 ± 0.1	<0.0001*
**Diabetes (n, %)**	8 (0.9%)	12 (4.3%)	20 (1.7%)	<0.0001*
**MetS (n, %)**	9 (1%)	31 (11.1%)	40 (3.4%)	<0.0001*

BMI = body-mass index, MetS = metabolic syndrome, M = mean, n = number, SD = standard deviation, WHR = waist-to-hip ratio.

*Statistical significance for p < 0.05.

In all fasting groups, subjects with non-alcoholic fatty liver had higher mean concentrations of *total cholesterol* (*1*–*4 h:* p = 0.0203; *4*–*8 h:* p < 0.0001; *>8 h:* p = 0.0022), *LDL cholesterol* (*1*–*4 h:* p = 0.0577; *4*–*8 h:* p < 0.0001; *> 8 h:* p = 0.0051) and *triglycerides* (*1*–*4 h:* p < 0.0001; *4–8 h:* p < 0.0001; *> 8 h:* p < 0.0001). By contrast, *HDL cholesterol* concentrations were, on average, lower in subjects with hepatic steatosis (*1*–*4 h:* p = 0.0055; *4*–*8 h:* p < 0.0001; *> 8 h:* p = 0.0045; Table 5). With the exception of *LDL cholesterol* in the group reporting 1–4 hours’ fasting, all differences were statistically significant.

**Table 5:** Mean concentrations of the lipid fractions in relation to fasting group and steatosis hepatis

	**Fasting time**						**p**
	**1-4 h**		**4-8 h**		**>8 h**		
	**n**	**M ± SD (%**^**§**^**)**	**n**	**M ± SD (%**^**§**^**)**	**n**	**M ± SD (%**^**§**^**)**	
**Total cholesterol**							
**No NAFLD**	118	5.2 ± 1.0 (−1.9%)	659	5.4 ± 1.0 (+1.9%)	139	5.3 ± 1.1 (Ref.)	0.0485*
**NAFLD**	33	5.6 ± 0.9 (−5.1%)	207	5.9 ± 1.1 (0%)	39	5.9 ± 1.1 (Ref.)	0.4354
**p**	-	0.0203*	-	<0.0001*	-	0.0022*	-
**LDL cholesterol**							
**No NAFLD**	118	2.9 ± 0.8 (−12.1%)	659	3.2 ± 0.9 (−3%)	139	3.3 ± 1.0 (Ref.)	0.0016*
**NAFLD**	33	3.2 ± 0.7 (−15.8%)	207	3.6 ± 1.0 (−5.3%)	39	3.8 ± 0.9 (Ref.)	0.0203*
**p**	-	0.0577	-	<0.0001*	-	0.0051*	-
**HDL cholesterol**							
**No NAFLD**	118	1.6 ± 0.4 (+6.7%)	659	1.7 ± 0.4 (+13.3%)	139	1.5 ± 0.4 (Ref.)	0.0004*
**NAFLD**	33	1.4 ± 0.4 (+7.7%)	207	1.4 ± 0.4 (+7.7%)	39	1,3 ± 0.4 (Ref.)	0.5016
**p**	-	0.0055*	-	<0.0001*	-	0.0045*	
**Triglycerides**							
**No NAFLD**	118	1.5 ± 0.9 (+50%)	659	1.3 ± 0.7 (+30%)	139	1.0 ± 0.5 (Ref.)	<0.0001*
**NAFLD**	33	2.3 ± 1.1 (+35.3%)	207	1.9 ± 1.0 (+11.8%)	39	1.7 ± 0.9 (Ref.)	0.0382*
**p**	-	<0.0001*	-	<0.0001*	-	<0.0001*	-

h = hour, HDL cholesterol = high-density lipoprotein cholesterol, LDL cholesterol = low-density lipoprotein cholesterol, M = Mean, n = number, Ref. = reference range, SD = standard deviation.

*Statistical significance for p < 0.05.

^§^Percent variability in comparison with the > 8 hour fasting group.

At multivariate analysis, however, hepatic steatosis was shown to be a significant independent factor for *elevated total cholesterol* (OR: 1.680, *95%-CI:* 1.058 - 2.669, p = 0.0278) and *elevated triglycerides* (OR: 2.350, *95%-CI:* 1.500 - 3.682, p = 0.0002; Table 6) only in males. Among females, no significant association was shown fatty liver as an independent factor for elevated lipid concentrations.

**Table 6:** Association between non-alcoholic fatty liver and elevated lipid concentrations

	**Women**		**Men**	
	**OR (95%-CI)**	**p**	**OR (95%-CI)**	**p**
**Total cholesterol**				
**NAFLD**	1.208 (0.647 - 2.257)	0.5530	1.680 (1.058 - 2.669)	0.0278*
**No NAFLD**	Ref.	-	Ref.	-
**LDL cholesterol**				
**NAFLD**	0.835 (0.453 - 1.540)	0.5634	1.309 (0.828 - 2.072)	0.2494
**No NAFLD**	Ref.	-	Ref.	-
**HDL cholesterol**				
**NAFLD**	2.207 (0.879 - 5.542)	0.0503	1.407 (0.805 - 2.459)	0.2310
**No NAFLD**	Ref.	-	Ref.	-
**Triglycerides**				
**NAFLD**	1.515 (0.784 - 2.926)	0.2166	2.350 (1.500 - 3.682)	0.0002*
**No NAFLD**	Ref.	-	Ref.	-

95%-CI = 95% confidence interval, h = hours, HDL cholesterol = high-density lipoprotein cholesterol, LDL cholesterol = low-density lipoprotein cholesterol, NAFLD = non-alcoholic fatty liver disease, OR = odds ratio, Ref. = reference range. *Significant association (p < 0.05).

## Discussion

### Lipid concentrations and fasting time

The determination of blood lipid levels in *fasting* and *non-fasting* subjects remains controversial [14-16]. Few studies have investigated this question in randomly selected population-based collectives. To our knowledge, only two published studies have examined the effect of fasting time on the plasma concentrations of *total cholesterol*, *HDL cholesterol*, *LDL cholesterol* and *triglycerides* in population-based collectives [2,8]. Sidhu et al., in a study of 209,180 subjects, observed that, with maximum variations of < 2%, total cholesterol and HDL cholesterol were least dependent on the pre-phlebotomy fasting time [2]. Langsted et al. analyzed the lipid concentrations of a total of 33,391 subjects in the *Copenhagen General Population Study* and the *Copenhagen City Heart Study:* they describe a reduction in average HDL and total cholesterol concentrations of no more than −0.1 and −0.2 mmol/l, respectively, following normal food intake [8]. Both studies had a comparable proportion of female subjects; however, the average age (52.8 and 60 years, respectively) was higher than that of the present collective. For total cholesterol, our data show maximum variability of 0% or 0 mmol/l in women and of −5.6% or −0.3 mmol/l in men. For HDL cholesterol, the maximum variability stands at +5.9% or +0.1 mmol/l for women and +7.7% or +0.1 mmol/l for men. Compared with the above cited studies, our data show wider variability of total cholesterol concentrations among male subjects and larger percentual variability of HDL concentrations in both sexes, though the absolute variability is identical to that reported by Langsted et al. With only 1,195 subjects, our collective was much smaller and, unlike the above cited studies, we did not break down our subject collective into subgroups according to *hourly* fasting intervals. Despite these differences in our study population compared with those of Sidhu et al. and Langsted et al., multivariate logistic regression analysis revealed no significant association between fasting time and elevated or debased total cholesterol and HDL cholesterol levels, which would indicate no substantial effect of pre- phlebotomy fasting.

The maximum range of the mean LDL cholesterol concentrations in the group reporting 1–4 hours’ fasting was −17.1% or −0.6 mmol/l in men and −6.1% or −0.2mmol/l in women. Thus, the distribution of the concentrations in men in the present study was greater than in the above-cited studies of Sidhu et al. and Langsted et al., which report on variability of < 10% or of no more than −0.2 mmol/l [2,8]. However, both Sidhu et al. and Langsted et al. observed the greatest variability of LDL cholesterol concentrations in the shorter fasting intervals of 1–4 h or 0–2 h following last food intake [2,8]. At multivariate logistic regression analysis we observed a significant association between fasting time and debased LDL cholesterol in men indicating that phlebotomy in the non-fasting state yields false low LDL cholesterol values. Several studies that observed the kinetics of lipoproteins in postprandial states showed that there is a decline in total cholesterol, HDL cholesterol and LDL cholesterol following food intake [4,17]. While Langsted et al. identified hemodilution as a plausible cause of decreased total cholesterol and LDL cholesterol values, they also showed that HDL cholesterol is actually decreased in the non-fasting state presumably due to bidirectional lipid exchange between HDL and triglyceride-rich lipoproteins, which are predominant in postprandial states [8]. However, as discussed above, these alterations of HDL cholesterol seem to be minimal.

Triglyceride concentrations showed a maximum variability of +66.7% or +0.6 mmol/l in women and +46.2% or +0.6 mmol/l in men, both in the group with the shortest pre-phlebotomy fasting period of 1–4 h. This exceeds the maximum variability reported in both sexes by Sidhu et al. and Langsted et al., who described a variability of < 20% or no more than +0.3 mmol/l. This may be due to the smaller number of subjects in both the total collective and in the group with the shortest reported fasting period of 1–4 h, to failure to exclude outliers and to differences in dividing the fasting intervals. A further potential factor could be the different prevalences of overweight and insulin resistance. In all fasting groups, men had significantly higher triglyceride concentrations than did women. This supports the observation that men experience a more pronounced postprandial spike in triglyceride concentrations than do women [12,18]. In agreement with the above-cited population-based studies, however, our results confirm that triglyceride concentrations are most dependent on the duration of the pre-phlebotomy fasting period and that this variability is most pronounced at early postprandial measurement. Based on these findings, measurement of triglycerides in *non-fasting* patients would seem unacceptable.

Considering atherogenesis as a postprandial phenomenon, *non-fasting* triglyceride concentrations could provide evidence regarding the concentrations to which the organism is exposed under normal, i.e. *non-fasting* conditions. For example, Eberly et al. reported that 19% of their subjects with a postprandial hypertriglyceridemia > 500 mg/dl showed fasting triglyceride concentrations of < 200 mg/dl [9]. These patients would have evaded detection with *fasting* triglyceride measurements alone. Disturbances of postprandial triglyceride clearance with very high and protracted plasma triglyceride concentrations have been described in persons with insulin resistance [12] and associated disorders, such as metabolic syndrome [19-22] and hepatic steatosis [23-25]. Hence, fasting measurement might lead to an inaccurate assessment of the ordinarily very predominant plasma triglyceride concentration in these patients.

### Limitations of the study

Several limitations of the present study must now be discussed. First, we had no information regarding the type and amount of food consumed by study subjects at their last pre-phlebotomy meal. These factors could adversely affect the results. Second, subjects self-reported their fasting times and we had no means of validating these reports. In addition, it was not possible to more precisely present fasting times in hourly intervals. Third, assignment of subjects on the basis of fasting time could not be matched in terms of number, age, sex and anthropometric criteria, leading to an unequal distribution of the number of subjects and their characteristics. Fourth, LDL cholesterol concentrations were indirectly calculated using the Friedewald formula in subjects with triglyceride concentrations < 4.6 mmol/l and absence of chylomicronemia. The calculated LDL cholesterol concentration tends to false-low results with triglyceride concentrations > 4.6 mmol/l [26], for which reason samples testing above this cut-off are measured directly. Use of the Friedewald formula may, however, also give false-low concentrations with LDL cholesterol concentrations < 2.0 - 3.0 mmol/l [27,28]. This parallel use of both calculated and measured concentrations in the present study may have introduced a further potential source of error. In subjects with type III hypercholesterolemia, the accumulation of cholesterol-rich lipoproteins results in erroneous high LDL cholesterol values calculated with the Friedewald formula [26,28]. The unknown prevalence of this rare metabolic disorder in the study collective could have resulted in distortion of the study findings. Finally, it might be presumed that the observed dependence of the LDL cholesterol concentration in subjects’ fasting time might be due, at least in part, that this concentration might be calculated from the triglyceride concentrations that are also dependent on fasting time. Langsted et al., however, have shown that the postprandial variability in the LDL cholesterol concentration is due to hemodilution [8]: thus, fasting time exerts an actual effect on LDL cholesterol concentrations. Fifth, the diagnosis of hepatic steatosis was made using ultrasonography. Thus, we had no information on the histological grade of the NAFLD. Sixth, data from our study collective showed pronounced scattering of lipid concentrations. Elimination of these outliers from the statistical analysis would have significantly reduced the size of our study collective.

## Conclusion

In conclusion, it can be stated that the lipid fractions *total cholesterol* and *HDL cholesterol* show slight, though partially statistically significant variability in relation to subjects’ fasting time; *triglycerides* and *LDL cholesterol* show the widest variability in relation to fasting times. In agreement with the recommendations of NCEP-ATP-III, which considers measurement of *HDL and total cholesterol* in non-fasting patients to be acceptable [1], our data showed no association between fasting time as an independent and pathological *HDL and total cholesterol concentrations* as a dependent variable. Whether measurement of *LDL cholesterol* and of *triglyceride* concentrations in *non-fasting* patient is reliable could not be definitively elucidated in the present study since both lipid fractions showed highly significant variability in relation to fasting time. In order to definitively answer this question, further large population-based studies with a representative subject collective are needed. In addition, further prospective studies must determine whether *non-fasting* lipid concentrations are comparably or better suited than *fasting* lipid profiles to predict metabolic diseases.

## Subjects and methods

### Study population

The EMIL Study (Echinococcus Multilocularis in Leutkirch) was conducted from November to December 2002 in Leutkirch, a town in southwestern Germany [29]. A total of 4,000 persons were randomly selected. Of these, 2,445 persons aged 10–65 years participated in the study (participation rate: 62.8%).

**Figure 1:**
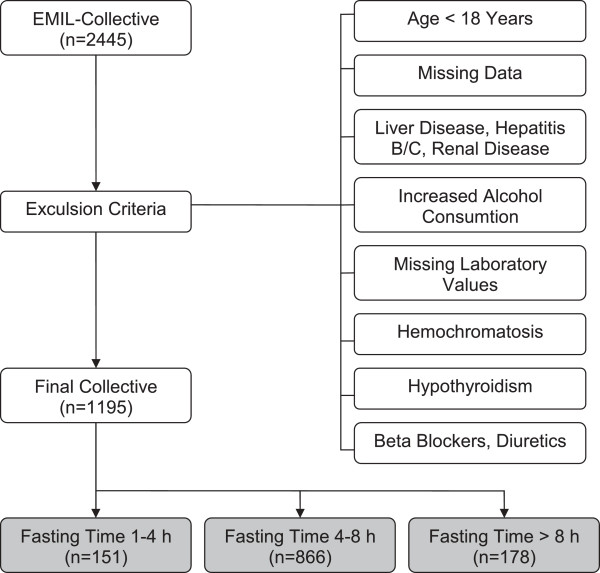
**Exclusion criteria and study population.** Use of beta blockers and diuretics was stated in the case history, increased alcohol consumption: females > 20 g/d, males > 40 g/d, h = hours, hypothyroidism: basal TSH > 4 mIU/Liter, n = number.

A total of 1,195 subjects remained after application of the following exclusion criteria for the study: subject age < 18 years; missing or incomplete data on history of liver diseases, hepatitis, gallbladder disorders, renal disorders, diabetes mellitus, arterial hypertension, dyslipidemia, tobacco smoking and duration of pre-phlebotomy fasting; positive history of liver diseases, hepatitis B and C, and chronic renal disorders; alcohol consumption > 20 g/day in women and > 40 g/day in men; missing relevant laboratory values (total cholesterol, triglycerides, HDL and LDL cholesterol, glucose, TSH, ALT, AST, GGT, AP); missing anthropometric data (BMI, WHR); pathological laboratory findings relative to hepatitis B and C, questionable hepatitis, hemochromatosis and hypothyroidism (basal TSH > 4 mIU/liter); pharmacological treatment with beta blockers or diuretics (see Figure 1).

The EMIL study was conducted in accordance with the Helsinki Declaration and Good Clinical Practice guidelines. Each subject provided informed written consent to participation in the study. The study was approved by the ethics commission of the State Medical Board of Baden-Württemberg (No. 133–02, 24 September 2002).

### Examination methods

Subjects’ medical history (including demographic data), recreational activities, medication history, family history, use of alcohol and tobacco, and dietary habits were documented using a standardized questionnaire.

### Duration of fasting period

At the time of phlebotomy, subjects were questioned regarding the time of their last food intake. Based on their responses, subjects were then assigned to one of three groups with fasting times of 1–4 hours, 4–8 hours and > 8 hours since last food intake.

### Anthropometric data

Body height, body weight, hip and waist circumference were measured. The body-mass index (BMI) and the waist-to-hip ratio (WHR) were calculated according to WHO recommendations [30].

### Laboratory analyses

Each subject underwent phlebotomy to obtain ca. 25 ml of venous whole blood from a cubital vein. Total cholesterol, triglycerides, LDL and HDL cholesterol concentrations were determined using a Dimension RxL unit (Dade-Behring, Germany). If triglyceride concentrations were < 4.6 mmol/liter and chylomicronemia was not reported, the *LDL cholesterol* concentration was calculated using the Friedewald formula [26]:

$$\begin{array}{l} \mathrm{LDL}\;\mathrm{cholesterol}=\left(\mathrm{Total}\;\mathrm{cholesterol}-\mathrm{triglycerides}\right)\\ \;/\left(2.2-\mathrm{HDL}\;\mathrm{cholesterol}\right) \end{array}$$

Lipid concentrations are given as mmol/liter (in order to convert HDL, LDL and total cholesterol concentrations to mg/dl, the molar value is multiplied by 38.67; for triglycerides, the multiplication factor is 87.5). Normal values were based on the reference range of the Department of Clinical Chemistry of the University of Ulm: total cholesterol < 5 mmol/l; LDL cholesterol < 3 mmol/l; HDL cholesterol > 1.2 mmol/l in women and > 1.0 mmol/l in men; triglycerides < 1.7 mmol/l.

### Definition of diabetes mellitus and the metabolic syndrome

Subjects were asked about a history of diabetes mellitus when completing the questionnaire. A diagnosis of metabolic syndrome was based on the NCEP-ATP-III criteria. Because not all subjects were fasting at the time of phlebotomy as required by the guidelines, the ATP-III criteria were modified as follows: based on the assumption that the blood glucose concentration should not exceed 160 mg/dl at the end of a 30-minute waiting time prior to phlebotomy, this concentration was established as the upper limit. In order to fulfill the definition for metabolic syndrome, subjects had therefore to meet at least three of the following criteria: waist circumference > 102 cm in males and > 88 cm in females; serum triglycerides > 1.7 mmol/liter; HDL cholesterol < 1.0 mmol/l in males and < 1.2 mmol/l in females; history of high-normal blood pressure readings (≥ 130/85 mmHg) or arterial hypertension (≥ 140/90 mmHg) or of anti-hypertensive medication; random blood glucose ≥ 160 mg/dl or history of diabetes mellitus.

### Ultrasonographic examination and criteria for hepatic steatosis

The ultrasonographic examinations were performed by specially trained examiners under standardized conditions using four identical HDI 5000 diagnostic ultrasound units (ATL Ultrasound, Philips Medical Systems, Bothell, WA, USA). Pathological or unclear findings were verified by an experienced supervisor (> 3,000 ultrasound examinations annually). As far as possible, initial settings were identical for the four units. Findings were recorded using a standardized report form. The liver, biliary tract and gallbladder, kidneys and spleen were examined. The liver was evaluated for size, and for presence of focal lesions and hepatic steatosis. Diagnostic criteria for hepatic steatosis are based on Saverymuttu et al., Hamaguchi et al. and Charatcharoenwitthaya et al. [31-33]: the hepatic parenchyma was compared with the renal parenchyma under consideration of the dorsal attenuation, visualization of the diaphragm and of the intrahepatic vessels. The severity of the steatosis was divided into four classes: no steatosis and mild (class I), moderate (class II) and severe (class III) steatosis.

### Statistical anaylsis

Statistical analysis of the data was performed using the SAS 9.2 statistics software (SAS Institute Inc., Cary, North Carolina, USA). Data were first analyzed descriptively. The mean and standard deviation were calculated for continuous variables. Categorical variables were represented with absolute and relative frequencies. In order to detect differences between the sexes and fastingtime groups, the Kruskal-Wallis test was used for continuous variables, while, for categorical variables, the chi-square test was used. Differences between the fasting time groups were identified using the Kruskal-Wallis test. The lipid factors total cholesterol, triglycerides, LDL and HDL cholesterol were tested for normal distribution as a continuous variable for the multivariate analyses. Testing revealed deviation from normal distribution. Logarithmic transformation also failed to demonstrate normal distribution. Therefore, the non-normally distributed lipid values were divided into normal and pathologic groups according to reference levels used in our clinic. Thefore, the influence of age, BMI, WHR, alcohol consumption, fasting time and hepatic steatosis on the respective lipid factors (total cholesterol, LDL and HDL cholesterol, triglycerides) was ascertained using multivariate logistic regression. Since the number of subjects meeting the criteria for diabetes mellitus (n = 20) or metabolic syndrome (n = 40) where too small (*also see* Table 1), these variables could not be considered using multivariate logistic regression. All tests were two-sided. Statistical significance was established at α = 5%. Values for *p* are given to the ten-thousandths decimal place.

## Abbreviations

μg: Microgram; ♀: Female; ♂: Male; 95%-CI: 95% confidence interval; ALT: Alanine aminotransferase; AP: Alkaline phosphatase; AST: Aspartate aminotransferase; BMI: Body-mass index; g: Gram; GGT: Gamma glutamyltransferase; h: Hours; HDL: High-density lipoprotein; LDL: Low-density lipoprotein; MetS: Metabolic syndrome; mg: Milligram; mIU/l: Milli international units per liter; M: Mean; n: Number; NAFLD: Non-alcoholic fatty liver disease; NCEP-ATP-III: National Cholesterol Education Program – Adult Treatment Panel III; OR: Odds ratio; Ref.: Reference range; SD: Standard deviation; TSH: Thyroid stimulating hormone; U: Units; WHO: World Health Organisation; WHR: Waist-to-hip ratio.

## Competing interests

The authors declare that they have no competing interests.

## Authors’ contributions

MG: Literature review, interpretation of data, writing of article. TG: Article revision, data analysis. SO: Study design, statistical analysis, interpretation of data. MH: Data compilation. WK (Wolfgang Koenig): Study design, revision. AI: Data compilation, study design. BB: Study design. RM: Article revision. WK (Wolfgang Kratzer): Study design, data compilation, article revision. AA: Data interpretation. All authors read and approved the final manuscript.
